# Social identity and racial disparities in science literacy

**DOI:** 10.1177/09636625221141378

**Published:** 2023-01-17

**Authors:** Kirils Makarovs, Nick Allum

**Affiliations:** University of Amsterdam, The Netherlands; University of Essex, UK

**Keywords:** public understanding of science, racial disparities, scientific literacy, social identity theory

## Abstract

Research on African-Americans’ relationship with science, while relatively sparse, in general suggests higher levels of alienation than among their White counterparts, whether in the form of less positive attitudes to science, or lower scientific literacy. In this article, we leverage social identity theory to examine the role of racial social identity and ingroup evaluation as putative mechanisms that produce these disparities. We use data from the General Social Survey, pooled over three waves, as the basis for our investigation. The results of the analysis indicate that, when controlling for other covariates, there is no statistically significant difference in the effect of racial self-identification on science knowledge among African-Americans and Whites. However, we provide evidence that the effect of favourable ingroup evaluation on science knowledge differs in these two groups, being more positive for African-Americans compared to Whites.

## 1. Introduction

The underrepresentation of Black Americans in STEM occupations, along with racial differences in educational experiences, lower levels of general literacy and restricted access to scientific information have been posited as important factors associated with racial disparities in knowledge about science (e.g. Anderson, 2015). Indeed, reports show that the share of Black Americans working in the field of science, technology, and engineering has been low at least since 1970 and continues to be so now (Landivar, 2013). Blacks are less likely to select STEM majors at college, and they have higher chances of dropping out (Chen, 2009; Griffith, 2010). In terms of schooling, Blacks’ overall experience also tend to be less positive than that of Whites (for a review see Kao and Thompson, 2003; see also (Fordham and Ogbu, 1986; Lynn and Parker, 2006). Concomitantly, levels of basic and health literacy for Blacks are lower than for other race and ethnic groups (NASEM 2016: Chapter 3; (Kutner et al., 2006; Rikard et al., 2016). It would be unsurprising, then, if science literacy followed the same pattern, plausibly also dependent on this common set of structural features.

Recent research suggests, however, that the racial cleavage in science knowledge is not only a mere reflection of broader patterns of social and economic disadvantage. Racial disparities in science knowledge persist even when people with the same educational levels are compared (Funk and Goo, 2015: 5; NSB 2018: 41–43). Adjusting for basic or ‘foundational’ literacy and a range of other covariates, Allum et al (2018) found that a substantial knowledge gap remains. This indicates that there may be something more at play than observably structural explanations for disparities in science knowledge. As Anderson (2015) notes, there arguably exists an historically established ‘complex relationship between science and the African-American community’. Blacks tend to be more anti-scientific in their attitudes (Gauchat, 2008) and have a lower level of trust in science (Gauchat, 2011, 2012). They also consider scientific misconduct to be a bigger issue compared to Whites, and this is especially so in the medical realm (Funk et al., 2019).

All of this is unsurprising: the legacy of ‘scientific racism’, as Plutzer puts it (2013: 147; see also Fairchild, 1991; Williams, 1974) is strong and may well drive some of the ways in which African-Americans perceive science. Apparent cases of science-driven discrimination (Dennis, 1995), such as Galton’s (1892) early work on eugenics and Jensen’s research on race-based differences in IQ, published in 1969, attracted widespread media attention (Sowell, 1973) and remains one of the most controversial scientific episodes of the 20th century. This and other famous cases such as that of the Tuskegee Syphilis Experiment (Fairchild and Bayer, 1999; e.g. Reverby, 2001) could have left a profound imprint on the collective memory of African-Americans (Assmann and Czaplicka, 1995; Halbwachs, 1992). This could quite reasonably give rise to suspicious – if not downright antagonistic – attitudes to science. This, in turn, could drive alienation and institutional distrust, and a lack of motivation to engage with science, including with formal and informal science education.

The perception that science does little for Black Americans is wryly captured in Gil Scott-Heron’s ironic paean to the space race of the 1960s: ‘The man jus’ upped my rent las’ night ‘cause Whitey’s on the moon. No hot water, no toilets, no lights. But Whitey’s on the moon’. For Scott-Heron, the fact that it is *White* Americans who are on the moon is significant. The salience of race in one of the most spectacular scientific achievements of the last century derives from the harsh contrast between the deprived material conditions experienced by Blacks at the end of the 1960s while seemingly unlimited federal resources were simultaneously being channelled to the space race. Thus, it is plausible that race-based social identity could be associated with attitudes to and engagement with science, if the social gains from science are seen as inequitably distributed along racial dimensions.

In this article, we take up this theme and adopt a social psychological approach based on social identity theory (SIT; Tajfel, 1974, 1981; Tajfel and Turner, 1986) along with the related idea of stereotype threat (Aronson, 2004). In doing so, we seek to elaborate on findings emerging from recent research by investigating how the salience of racial-identification and ingroup evaluation might be connected with disparities in science literacy.

## 2. Theoretical framework and hypotheses

### Social Identity Theory

The cornerstone concept of SIT is, unsurprisingly, social identity. Produced by a process of social categorization, which implies systematizing the social world according to meaningful and distinct categories, social identity describes the state of one’s belonging to a certain social group and the meanings that this belonging entails. According to Tajfel, social identity is ‘that part of an individual’s self-concept which derives from his knowledge of his membership in a social group (or groups) together with the value and emotional significance attached to that group membership’ (Tajfel, 1981: 255).

A subjective interpretation of a group membership is implied by SIT and the concept of a social group is regarded as flexible as well, being treated as ‘a cognitive entity that is meaningful to the subject at a particular point in time’ (Tajfel, 1974: 69). Therefore, it should not be confused with sociological categories which imply an external, observer-driven categorization of social objects (Turner and Reynolds, 2001: 137–138). The core mechanism implied by the theory, namely, dividing people into ingroup and outgroup, brings about three theoretical principles underpinning the dynamics of intergroup behaviour: (a) the desire to maintain a positive social identity; (b) fulfilment of this desire by making a favourable comparison with a relevant outgroup, and (c) leaving, or changing the value of the social group, if the social identity provided by it appears to be unsatisfactory (Tajfel and Turner, 1986: 16). However, not every identity is equally important. The concept of ‘master statuses’ (Jaret and Reitzes, 1999: 716–717; see Rosenblum and Travis, 1996) refers to those substantial characteristics (race, gender, class, and sexual orientation are examples) that overwhelm other identities in structuring social situations. Racial identity is arguably the most pivotal among them, since it is rarely possible to mask one’s phenotypical traits that are used by others in a process of categorization and thus escape or alter its consequences.^1^

While Whites’ racial identity is stereotypically associated with being more educated (Allen, 1996) and having higher social status (Saperstein and Penner, 2012), Blacks are oftentimes subjected to negative prejudices about their behaviour and intellectual abilities (e.g. Peffley et al., 1997). Even though the awareness of such negative stereotypes could in principle lead to enhanced social solidarity, the need constantly to refute unfounded allegations can lead to a substantial decrease in well-being (Hughes et al., 2015) and ultimately result in the internalization of negative racial stereotypes and a distorted view of oneself and one’s abilities (Williams and Mohammed, 2013). Our intuition is that science is seen as alienating for at least some Black Americans (this is not saying that it may not be alienating for some Whites too). That being so, it is reasonable to suggest that variation in the salience of racial identity for Americans could shape some of the variation in their attitudes and knowledge in relation to science. Accordingly, our first research question is:

*How is the salience of racial self-identification associated with science literacy for Blacks compared to Whites?*

### Racial divide in the salience of racial self-identification

There is substantial empirical evidence to suggest that racial self-identification plays an essential role in structuring the everyday life of African-Americans and that it is less salient among Whites. Distinctiveness theory suggests a plausible explanation for this fact, arguing that self-identities based on traits that readily distinguish a person from others around them tend to be more salient than those that do not (Mehra et al., 1998). Hence, African-Americans that make up a visible racial minority are more likely than Whites to embrace racial self-identification as a crucial component of their social identity. This is consistent with findings from survey research. Blacks, on average, report feeling closer to the people of their race (Thornton et al., 2012; Williams et al., 2012; Wong 1998; Wong and Cho, 2005) and are more likely to mention race as an identity that is ‘most important to you in describing who you are’ (Smith, 2007: 388). This feeling of overall closeness translates into the acknowledgement of common history and common fate (Bobo and Johnson, 2000: 95) which, in turn, gives ground for race-based political engagement (e.g. Gurin et al., 1990; Tate, 1994).

Not only do Blacks tend to *feel* that they are united with other Blacks but this perceived social closeness is also intertwined with long-term socioeconomic conditions. Racial disparities perpetuate in a host of different ways, for instance, in terms of place of living (Emerson et al., 2001; Iceland and Weinberg, 2002), studying (Goldsmith, 2009; Roscigno, 1998) and strategies for finding a job (Mouw, 2002).

Whites, on the contrary, tend to put less emphasis on their racial belonging (Croll, 2007; Wong and Cho, 2005). Being a dominant racial identity in the United States, Whiteness serves as the ‘unmarked norm against which other identities are marked and racialized’ (Rasmussen et al., 2001). While being barely noticeable to Whites themselves, White racial self-identification can be an object of aspiration and is linked with the achievement of higher social status (Saperstein and Penner, 2012; Telles and Paschel, 2014).

In this way, given that the racial identity is more prominent among Blacks than among Whites, and recognizing that the premises for science alienation could be entrenched in Black racial self-identification, we hypothesize that, *for Black Americans, stronger racial self-identification will be associated with lower levels of civic scientific literacy than for Whites. (Hypothesis A).*

### Ingroup evaluation

Our second research question focuses more specifically on ingroup evaluation as a vital part of the self-identification process and asks *how ingroup evaluation is associated with science literacy for Blacks compared to how it is associated for Whites.* We explain the rationale for asking this question in what follows.

Retaining a positive social identity is an important task for an individual, and there are several options for doing so, according to SIT. The most common way is to make a favourable comparison with a relevant outgroup. One can also abandon a social group that has a lower status (Ellemers and Haslam, 2011; Tajfel and Turner, 1986). Since changing one’s racial identity is quite problematic because of the hardly permeable borders dividing racial identities (Hughes et al., 2015: 28), emphasizing the distinctiveness of one’s own racial group and amplifying its advantages over the outgroup can become a common practice to maintain a positive identity for members of devalued groups.

This social mechanism of raising collective self-esteem (Crocker and Luhtanen, 1990) that manifests itself in accentuating one’s distinctiveness, for example, by celebrating race-specific cultural heritage (Tajfel, 1974: 83) is likely especially vital for Black Americans: as their self-identification is very much based on repelling identity-threatening stereotypes. Allport (1954) noted that African Americans ‘have heard so frequently that they are lazy, ignorant, dirty, and superstitious that they may half believe the accusations, and since the traits are commonly despised. . . some degree of in-group hate seems almost inevitable’ (p. 152, cited by Burkley and Blanton, 2009: 287). While this was in the context of the Jim Crow America of the 1950s, there is little reason to think that things have changed radically in the intervening years.

Positive ingroup evaluation and even ingroup bias, as a radical form of favourable ingroup comparison (Kiecolt and Hughes, 2017; Rudman et al., 2002), does not imply that Blacks should necessarily endorse a positive cultural notion of science per se, but it could nevertheless serve as a ground for resisting a stereotype threat. Stereotype threat is a widely studied socio-psychological phenomenon that provides insight into how self-identification interacting with commonly held stereotypes might influence one’s actions and worsen performance in the area which is subject to the stereotyping (Aronson, 2004; Steele, 1997). Social-psychological experiments (e.g. Steele and Aronson, 1995) have shown that African-American students underperform considerably compared to Whites in a verbal test when it is framed as a test of abilities, rather than one exploring general psychological factors. Presumably, the need to confront negative societal stereotypes about their intellectual abilities is what puts on them ‘an extra cognitive and emotional burden not borne by people for whom the stereotype does not apply’ (Aronson et al., 2002: 114), resulting in more stress and weakened performance. Salient racial self-identification, in this regard, can play the role of catalyst making African-Americans to internalize more deeply the negative racial stereotypes (Armenta, 2010; e.g. Shih et al., 1999).

In contradistinction to this tendency, those that hold positive outlooks about members of their racial ingroup will be more likely to question and resist racial intelligence stereotypes. This in turn may mitigate their negative effect on performance (Aronson et al., 2002). Thus, treating a positive ingroup evaluation as a signal that the individual’s level of ‘inferior anxiety’ (Steele and Aronson, 1995: 797–798) is reduced and that they are less subjected to, or at least affected by, a stereotypical notion of intellectual capacities throughout the life course, we expect to see that *for Black Americans, higher levels of positive ingroup evaluation will be associated with higher levels of civic scientific literacy than for Whites. (Hypothesis B).*

## 3. Data, measures and analytical strategy

### Data

Data for this study come from the General Social Survey (GSS), which is a biennial, face-to-face probability survey of the adult population of the United States covering a wide range of social and political attitudes and beliefs, including racial identity. The GSS has also featured measures of science literacy since 2006. The variables required for our analysis are only found together in the same questionnaire version in three of the survey years available (2008, 2010, 2016, see supplemental material). We therefore combine respondents from all of them into one data set. The response rates were 70.4% and 70.3% in 2008 and 2010, respectively, and 61.3% in 2016.^2^ Survey weights were applied in the regression modelling to account for an equal-probability multi-stage cluster sampling design of the GSS.

### Measures

Following the literature on civic scientific literacy (Allum et al., 2008; Miller, 1987, 1998, 2010, 2016), *science literacy* was measured as a number of correct (‘True’ or ‘False’) answers to a set of 14 quiz-type questions examining respondents’ knowledge of basic scientific facts, the idea of probability and the principles of experimental research (see also Allum et al., 2018; Gauchat, 2012). ‘Don’t know’ and refusals were treated as wrong answers. The list of items used to comprise this variable along with correct responses is presented in supplemental material.

Respondents’ *race* was measured with a dummy variable, indicating whether a person is White or Black. In this question, the interviewer was asked to code respondent’s race silently and ask a direct question only in the case of doubt. Those who fell into the category of ‘other race’ were omitted from the analysis because the heterogeneity within this category makes it impossible to capture the salience of a specific racial identity.^3^

*Racial self-identification* and *ingroup evaluation* were measured in a variety of ways (for a review see Wong and Cho, 2005: 703). In our case, we use five items included in the GSS to capture how strongly one associates oneself with one’s race ingroup and how one evaluates the members of the ingroup. Item wordings, response scales, and some examples of previous use of these items, are shown in Table 1.

**Table 1.** Measures of racial self-identification and ingroup evaluation.^a^

Question wording	Scale	Some examples of usage
In general, how close do you feel to Blacks/Whites? (*close*)	1 (Not at all close) to 9 (Very close)	Hughes and Tuch (2003) as a measure of social distance Kiecolt and Hughes (2017) as an indicator of racial self-identification
What about having a close relative marry a Black/White person? (*mar*)	1 (Strongly oppose) to 5 (Strongly favour)	St. Jean (1998); Djamba and Kimuna (2014) as an attitude to marriage outside own race. Barkan and Cohn (1994) as a measure of racial prejudice
What about living in a neighbourhood where half of your neighbours were Blacks/Whites? (*live*)	1 (Strongly oppose) to 5 (Strongly favour)	Weaver (2008) as a measure of social distance Barkan and Cohn (1994) as a measure of racial prejudice
The second set of characteristics asks if people in the group tend to be hardworking or if they tend to be lazy. Where would you rate Blacks/Whites in general on this scale? (*work*)	1 (Lazy) to 7 (Hardworking)	Kiecolt and Hughes (2017); Hughes et al (2015) as measures of racial ingroup evaluation
Do people in these groups tend to be unintelligent or tend to be intelligent? Where would you rate Blacks/Whites in general on this scale? (*intl*)	1 (Unintelligent) to 7 (Intelligent)	

a In the questionnaire there are 10 questions, as each of those items in the table was asked separately of all respondents about both Blacks and Whites. For the purposes of this analysis, they were recoded and the answers in respect of the other race category were discarded. This means that, for each of the five items, Black respondents’ answers are about Black people and Whites’ answers about Whites, in order that they can be interpreted as measures of self-identification and ingroup evaluation.

In order to investigate the latent nature of these concepts, an exploratory factor analysis was conducted on the five standardized items. A two-factor model with oblique rotation (Promax) yielded the most comprehensible result (see supplemental material). The first factor is related to the variables touching upon the issue of social distance (interracial marriage and composition of neighbourhood), thus indicating the measure of the salience of racial self-identification, while the second one is mainly composed of variables exploring capacities (industriousness and intelligence) of peer ingroup members, indicating the overall ingroup evaluation. The item referring to the general estimation of racial affinity (*close*) was almost equally explained by both factors. Factor score estimates were saved and used as independent variables in further analysis. The mean score of racial self-identification is −0.05 (sd = 0.75) for Whites and 0.06 (sd = 0.80) for Blacks. The mean score of racial ingroup evaluation is −0.03 (sd = 0.66) for Whites and −0.04 (sd = 0.77) for Blacks.

We also employ three variables that previously have been suggested as potential confounders on science literacy–respondent’s *level of education*, *participation in college science courses*, and *foundational literacy*. Those having a college degree and taking science courses while studying generally tend to be more knowledgeable in science (Funk and Goo, 2015: 4; Miller, 2010; NSB 2018: 37; Plutzer, 2013). An examination of the relationship between foundational literacy, using the same variable as we do here (*wordsum*, a vocabulary test administered to all GSS respondents) and science knowledge was carried out by Allum et al. (2018), who found that the inclusion of foundational literacy accounted for part of the covariance between race and science literacy. Hence, we include it in our analyses. We also adjust for *religiosity*, *family income*, *age*, *gender*, and *political affiliation*. Details of all these covariates are shown in Table 2.

**Table 2.** Descriptive statistics of variables used in the analysis, n = 1300.

Variable		Mean	SD	Min	Max
Civic scientific literacy	*R*’s score on a science knowledge quiz	8.81	2.78	1	14
White	Whether *R* is White (1 = Yes, 0 = No)	0.83		0	1
Racial self-identification	Factor 1 saved scores	–0.02	0.75	–2.85	1.51
Ingroup evaluation	Factor 2 saved scores	–0.04	0.66	–2.54	2.08
College-level science courses	Whether *R* has taken any college-level science courses (1 = Yes, 0 = No)	0.43		0	1
Education	Highest year of school completed	13.78	2.79	0	20
Female	Whether *R* is female (1 = Yes, 0 = No)	0.56		0	1
Age	*R*’s age	47.99	17.05	18	89
Foundational literacy	Total number of correct answers on a Wordsum vocabulary test	6.15	1.83	0	10
Church attendance	How often *R* attends religious services (0 = Never, 8 = More than once a week)	3.50	2.80	0	8
Independent	Whether *R* identifies as Independent, Independent, near Democrat, or Independent, near Republican (1 = Yes, 0 = No)	0.40		0	1
Republican	Whether *R* identifies as not strong or strong Republican (1 = Yes, 0 = No)	0.27		0	1
Family income	*R*’s inflation-adjusted family income, standardized	0.01	0.95	–1.13	2.93

SD: standard deviation.

Baseline race is Black and those categorized as ‘other’ were omitted from the analysis. Baseline political preference is Democrat (not strong or strong), and those affiliating themselves with ‘other party’ were omitted from the analysis. The number of observations corresponds to the fully specified regression models (Table 3, Models 7 and 8).

### Analytical strategy

In order to test our hypotheses, we fit a set of multivariate linear regressions with interaction terms. The interaction terms of race, with racial self-identification and ingroup evaluation, respectively, allow us to establish whether the effects of these two variables on science knowledge differ for Whites and Blacks. We expect that racial self-identification and ingroup evaluation have significantly different associations for Blacks than for Whites, who, in this regard, might be considered as a baseline for a comparison. We begin with models that predict science knowledge from the set of control variables and education-related covariates. The purpose here is to assess the magnitude of the racial disparity in science literacy, which is our explanandum in the models that follow. We then examine the zero-order relationships between the identity variables and science knowledge, without controls and interactions, before presenting fully specified models with all covariates included.

### Results

Table 3 presents parameter estimates for the models outlined above. The first model with controls only indicates that Whites, on average, tend to score almost two points higher on the science knowledge scale than Blacks, which is consistent with previous findings (Allum et al., 2018). Women, non-Democrats, older and more religious people have also, on average, poorer science knowledge, while higher family income is associated with higher knowledge. Model 2 adds covariates that account for the various facets of relevant educational and cognitive achievement. Having more years of schooling, undertaking at least some science-related college courses, and having a higher level of foundational literacy are all positively related to science knowledge. When these variables are accounted for, the association with race diminishes by one-third, family income drops by more than a half, and the association with political affiliation becomes small and insignificant.

**Table 3.** Parameter estimates for the models on civic scientific literacy.^a^

	Civic scientific literacy							
	(1)	(2)	(3)	(4)	(5)	(6)	(7)	(8)
Race and controls								
White	1.905*** (0.116)	1.283*** (0.135)	2.048*** (0.237)	2.065*** (0.238)	1.687*** (0.228)	1.694*** (0.228)	1.366*** (0.241)	1.365*** (0.239)
Female	–0.660*** (0.079)	–0.837*** (0.089)			–0.626*** (0.156)	–0.642*** (0.157)	–0.777*** (0.152)	–0.789*** (0.153)
Age	–0.026*** (0.002)	–0.026*** (0.002)			–0.024*** (0.004)	–0.024*** (0.004)	–0.024*** (0.004)	–0.024*** (0.004)
Independent	–0.212** (0.088)	–0.099 (0.093)			–0.156 (0.172)	–0.152 (0.169)	–0.088 (0.162)	–0.094 (0.159)
Republican	–0.207* (0.110)	–0.034 (0.125)			0.107 (0.211)	0.070 (0.212)	0.036 (0.192)	0.005 (0.190)
Family income	0.682*** (0.042)	0.184*** (0.047)			0.625*** (0.072)	0.608*** (0.072)	0.206*** (0.073)	0.199*** (0.073)
Church attendance	–0.084*** (0.015)	–0.099*** (0.017)			–0.107*** (0.032)	–0.109*** (0.031)	–0.098*** (0.028)	–0.099*** (0.028)
Education-related								
Education		0.157*** (0.022)					0.181*** (0.035)	0.180*** (0.035)
College-level science courses taken		0.714*** (0.125)					0.526*** (0.195)	0.533*** (0.196)
Foundational literacy		0.444*** (0.025)					0.427*** (0.047)	0.428*** (0.047)
Interaction terms								
Racial self-identification			–0.114 (0.260)		–0.022 (0.264)		–0.003 (0.256)	
White x Racial self-identification			–0.388 (0.280)		–0.369 (0.292)		–0.222 (0.277)	
Ingroup evaluation				–0.123 (0.261)		0.256 (0.242)		0.296 (0.240)
White × Ingroup evaluation				–0.523* (0.294)		–0.668** (0.272)		–0.534** (0.263)
Constant	9.186*** (0.155)	4.760*** (0.316)	6.953*** (0.220)	6.945*** (0.221)	9.108*** (0.321)	9.153*** (0.324)	4.292*** (0.525)	4.344*** (0.524)
Observations	6153	3720	1620	1620	1421	1421	1300	1300
Log likelihood	–14,999.790	–8445.311	–4007.740	–4004.421	–3410.107	–3410.441	–2,917.731	–2916.999
Akaike Inf. Crit.	30,015.580	16,912.620	8,023.479	8,016.842	6,840.215	6,840.883	5,861.462	5,859.997

Design-corrected standard errors reported in parentheses. Black is a reference category for race. Male is a reference category for gender. Democrat is a reference category for political preference.

a The difference in the number of observations per each model is due to the number of ballots available for each combination of variables. A multiple imputation procedure was conducted using the *R* package Amelia (Honaker et al., 2011) to see whether the results of the regression modelling in the full specification models (7, 8) are held when the loss in observations caused by missings in controls and educational variables is compensated for. Although the magnitude of the effect varies, it does not change the very patterns of the relationships between variables.

* *p* < 0.1; **p < 0.05; ***p < 0.01.

Models 3 and 4 provide the first direct look at our hypotheses. Both interaction terms go in the same direction, yet they are not significant at the 5% level. Model 3 shows that for Blacks, a one-unit increase (which is approximately one standard deviation) in the level of racial self-identification is associated with a −0.114 decrease in civic scientific literacy. For Whites, the interaction term is negative, and the slope therefore becomes even more negative overall. A similar pattern is recognizable in Model 4. For Blacks, a one-unit increase in the level of ingroup evaluation is associated with a −0.123 reduction in science literacy, whereas for Whites the decrease in literacy is even steeper for the same one-unit change in their ingroup evaluation.

Models 5 and 6 build upon the previous models by combining controls with interaction terms. This changes the picture somewhat. The same pattern is visible in Model 5 as in Model 3, with the coefficients being all attenuated and non-significant. Whites who identify more strongly as White, score less well on science knowledge, while for Blacks the effect of racial identification approaches zero. However, for racial ingroup evaluation, the effect sizes are greater than in the model with no controls. The slope for Blacks is positive and equals 0.256, while for Whites it remains negative (i.e. 0.256–0.668 = –0.412).

Models 7 and 8 are full-specification models combining controls, educational variables, and interaction terms. While a slight diminution in the magnitude of interaction coefficients compared to models 5 and 6 is noticeable, the principal relationships remain the same. Whatever the racial discrepancy in the effect of racial self-identification might be, it remains insignificant in Model 7, and Model 8 continues to show a negative slope of ingroup evaluation for Whites and a positive slope for Blacks.

**Figure 1.**
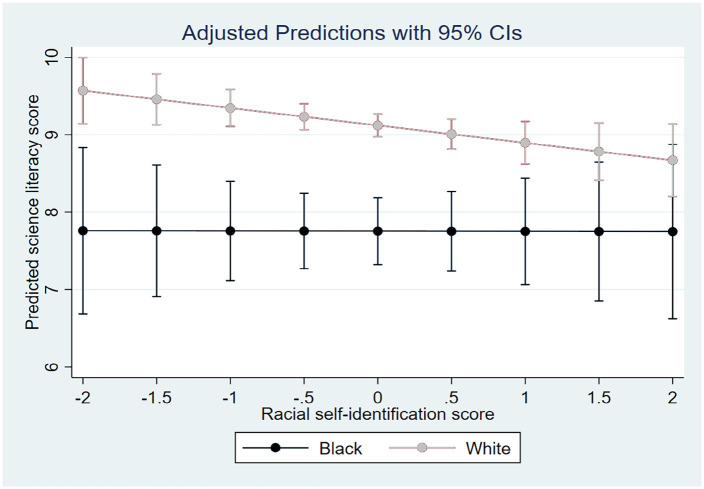
Predicted science literacy scores for Blacks and Whites across the values of racial self-identification. Corresponding to Model 7 in Table 3.

**Figure 2.**
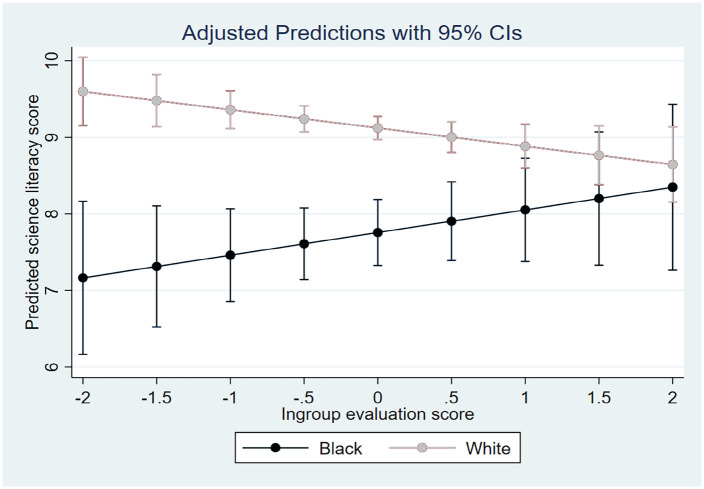
Predicted science literacy scores for Blacks and Whites across the values of ingroup evaluation. Corresponding to Model 8 in Table 3.

Figures 1 and 2 correspond to Models 7 and 8 and present the predicted science literacy scores for Blacks and Whites at various levels of racial self-identification and ingroup evaluation, respectively. Figure 1 shows that as the values of racial self-identification increase, the gap between Blacks and Whites in their predicted science literacy scores tends to diminish, yet insignificantly. On the contrary, Figure 2 reports that the higher the values of ingroup evaluation are, the less of the gap in predicted science literacy scores remains between Blacks and Whites, and this effect is statistically significant.

To summarize, no model has suggested that for Blacks more salient racial self-identification might lead to a lower level of civic scientific literacy than for Whites, which leads us broadly to rejecting hypothesis A. In fact, the models of interest (3, 5, 7) point out that the association of racial self-identification and science literacy for Whites seems to be more negative than for Blacks. Regarding hypothesis B, the situation is less ambiguous. None of our models have been able to show a higher level of ingroup evaluation associated with poorer knowledge of science for Blacks compared to Whites. In fact, the more positively the African-Americans evaluate their ingroup, the higher tends to be their observed level of science knowledge, and the smaller becomes the difference in predicted scores between them and Whites, for whom higher ingroup evaluation leads to lower science literacy scores. We regard hypothesis B, therefore, as receiving some support.

## 4. Discussion

This research derives its motivation from the idea that racial disparities in scientific literacy can be explained by introducing into the analysis the notion that differing attachments to racial identity interact with culturally fashioned and transmitted perceptions of science. Allum et al. (2018) highlighted ethnic and racial disparities in science literacy and that the way race predicts scores on a science knowledge scale is partially mediated by factors such as education and foundational literacy. In this study, we replicate this finding. We add to the discussion by showing that how one assesses the traits of one’s racial ingroup has different associations with scientific literacy for White and Black Americans. While these differential associations cannot account for the overall disparity between these groups, they suggest that for Blacks the in-group evaluation is something that is more positively linked to greater science literacy compared to Whites.

Contrary to our expectations, there is little evidence that the salience of racial identity itself is associated with lower scientific literacy among Blacks and, on the basis of our analysis and the variables used to operationalize racial self-identification, it cannot be regarded as a plausible explanation for observed disparities between White and Black Americans. While it is impossible to deny the historical trace of ‘scientific racism’ (Fairchild, 1991; Plutzer, 2013) affecting the well-being of racial and ethnic minorities in America, perhaps one might venture that the narratives perpetuating this malevolent experience are not as pronounced within the collective memory of African-Americans as might have been expected.

An alternative explanation might be due to ‘stereotype lift’ (Walton and Cohen, 2003) – the psychological mechanism which could in theory counterbalance the negative prejudice about science among Blacks. For some African-Americans, the stereotypical notion of a Black person who cannot be knowledgeable in science to the same degree as a White American might serve as a motivation for enhanced test performance (although it is fair to say that a knowledge quiz administered on the door-step is a low stakes test). Resisting the ‘chronic internalization’ (Burkley and Blanton, 2009: 287) of negative stereotypes about the ingroup and using social stigma as a self-protective mechanism (Crocker and Major, 1989) could in principle be boosting Black Americans’ interest in science and facilitating their uptake of scientific knowledge. This is also in line with the plentiful research showing that greater salience of racial identity brings more awareness about racial discrimination (e.g. Operario and Fiske, 2001; Sellers and Shelton, 2003; Shelton and Sellers, 2000), thus prompting people to find ways to bypass such prejudice. This idea, however, requires further empirical scrutiny. Observing the ‘flat’ effect of racial self-identification on science literacy for Blacks might encourage future research to replicate our results by using other, more precise measures of racial identity.

We found support for the idea that favourable ingroup evaluation is associated with higher science literacy for Black Americans compared to Whites. The positive evaluation of an ingroup that is generally stereotyped as less knowledgeable and intelligent could mean that for some Black Americans these stereotypes do not play a defining role in self-perception and in fact defying or ignoring these stereotypes through boosting ingroup evaluation can alleviate the burden of stereotype threat (Armenta, 2010; Aronson et al., 2002) and open the way to more engagement with science and concomitantly greater knowledge. The degree to which positive ingroup evaluation among minorities encourages engagement with science is an area in which more research could be directed.

Another notable finding, about which we had no firm prior expectations, is that increased salience of racial identity and positive ingroup evaluation are both associated with lower science literacy for White Americans. While the research on White racial identity suggests that it tends to be particularly strong among less educated males (Croll, 2007) and flourish in poor socio-economic environments (Oliver and Mendelberg, 2000), our study suggests that the science literacy of Whites is negatively associated with their racial identification, even when various facets of educational attainment and income level are taken into account. We might speculate here that increased identification with the dominant racial group perhaps stands as a proxy for more parochial, less cosmopolitan values. Science is arguably an inherently cosmopolitan enterprise and scientific knowledge may sit uneasily with a rather blinkered mind-set praising one’s belonging to a dominant racial group. This is consistent with Croll’s (2007) observation that White racial identity is especially salient among those who reject multiculturalism and believe that the Unites States is, or should be, a White nation.

In this article, we were able to leverage high-quality survey data to explore the association between identity, race and science literacy. However, surveys often provide a broad but shallow basis for inference. In the present case, one of the limitations is that the questions on identity in the GSS are very general in scope. It is possible that questions tapping identity-salience more directly linked to scientific issues may yield different results. For instance Dawson (2018) suggests that marginalized social groups do not express firm lack of interest in science; rather, the underlying reasons for their disengagement should be sought in the way in which the structure of scientific discourse per se provokes their perceived powerlessness and the feeling of inferiority to the present cultural order, ultimately excluding them from crucial science practices and limiting their ability to be heard, and perhaps to listen, too. At all events, there is much more to be learned about the basis of disparities in science literacy and an urgent societal need that they be diminished.

## Supplemental Material

sj-pdf-1-pus-10.1177_09636625221141378 – Supplemental material for Social identity and racial disparities in science literacy Click here for additional data file. Supplemental material, sj-pdf-1-pus-10.1177_09636625221141378 for Social identity and racial disparities in science literacy by Kirils Makarovs and Nick Allum in Public Understanding of Science

## Notes

1. Although racial self-identification might be a subject to fluidity and impermanence (see Harris and Jeremiah, 2002; Mowen and Stansfield, 2016; Telles and Paschel, 2014), this is more common for those having mixed ancestry or multiracial parents, and usually does not affect most of population, whose racial self-identification tends to be stable over time. However, see Saperstein and Penner (2012) on how self-classification and categorization by others are deeply intertwined with individuals’ social status.

2.
http://gss.norc.org/Documents/other/Response%20rates.pdf

3. Another way to ask about a race in a survey is to refer to respondents’ self-categorization. In General Social Survey (GSS), this option is provided by a variable *racecen1*, which counts a first mention on the following question: ‘What is your race? Indicate one or more races that you consider yourself to be’. While studies report that there may exist a gap between how an interviewer and oneself can see one’s racial belonging (Saperstein and Penner, 2014), this is not the case in the present analysis. Cross-tabulation shows that 99% of Whites or Blacks identified as such by an interviewer considered themselves as Whites or Blacks, respectively, when asked about their racial self-categorization.
