# Supplementary material for: Social identity and racial disparities in science literacy
Source: Public Underst Sci. 2023 Jan 17;32(3):373–88. doi: 10.1177/09636625221141378 (PMC10074749; doi:10.1177/09636625221141378)
Supplement: sj-pdf-1-pus-10.1177_09636625221141378 – Supplemental material for Social identity and racial disparities in science literacy [file sj-pdf-1-pus-10.1177_09636625221141378.pdf]

Supplemental material

Social identity and racial disparities in science literacy

Kirils Makarovs and Nick Allum

Table of Contents

S1. Ballot distribution of the items of interest, GSS 2006-2016.. 2

S2. The list of items used to construct the civic scientific literacy scale 3

S3. EFA standardized loadings, n = 1,620 4

S4. Regression models - Without evolved and bigbang items... 5

S5. Regression models – Only among Whites 8

S6. Regression models – Only among Blacks 10

S7. Regression models - Including science attitudes as a predictor 12

S8. Regression models - Including squared terms 14

S9. Regression models – Using the Difference measure of racial self-identification and ingroup-evaluation*.. 17

S1. Ballot distribution of the items of interest, GSS 2006-2016

Variable/Year	2006	2008	2010	2012	2014	2016
			Controls			
sex	A B C D	A B C	A B C	A B C	A B C	A B C
age	A B C D	A B C	A B C	A B C	A B C	A B C
partyid	A B C D	A B C	A B C	A B C	A B C	A B C
coninc	A B C D	A B C	A B C	A B C	A B C	A B C
attend	A B C D	A B C	A B C	A B C	A B C	A B C
			Civic scientific literacy composites			
hotcore	B C	A B C	A C	B C	B C	A B
radioact	B C	A B C	A C	B C	B C	A B
boyorgrl	A B C	A B C	A C	B C	B C	A B
lasers	B C	A B C	A C	B C	B C	A B
electron	B C	A B C	A C	B C	B C	A B
bigbang	B C	A B C	A C	B C	B C	A
condrift	B C	A B C	A C	B C	B C	A B
evolved	B C	A B C	A C	B C	B C	A B
earthsun	B C	A B C	A C	B C	B C	A B
solarrev	B C	A B C	A B C	B C	B C	A B
odds1	B C	A B C	A C	B C	B C	A B
odds2	B C	A B C	A C	B C	B C	A B
expdesgn	B C	A B C	A C	B C	B C	A B
			Education-related			
colsci	B C	A B C	A C	B C	B C	A B
wordsum	A B C	A B	A B C	A B	A B	A B
educ	A B C D	A B C	A B C	A B C	A B C	A B C
			Race-related			
race	A B C D	A B C	A B C	A B C	A B C	A B C
closeblk	A C	A C	A C	A C	A C	A C
closewht	A C	A C	A C	A C	A C	A C
marblk	A B	A B	A B	A B	A B	A B
marwht	A B	A B	A B	A B	A B	A B
liveblks	A B	A B	A B	A B	A B	A B
livewhts	A B	A B	A B	A B	A B	A B
workblks	A B	A B	A B	A B	A B	A B
workwhts	A B	A B	A B	A B	A B	A B
intlblks	A B	A B	A B	A B	A B	A B
intlwhts	A B	A B	A B	A B	A B	A B

Note: The information is taken from the General Social Survey Data Explorer available at: https://gssdataexplorer.norc.org/. The GSS survey year and ballots including all the relevant variables and used in the analysis are highlighted in green.

S2. The list of items used to construct the civic scientific literacy scale

1. The center of the Earth is very hot. (True)
2. All radioactivity is man-made. (False)
3. It is the father's gene that decides whether the baby is a boy or a girl. (True)
4. Lasers work by focusing sound waves. (False)
5. Electrons are smaller than atoms. (True)
6. Antibiotics kill viruses as well as bacteria. (False)
7. The universe began with a huge explosion. (True)
8. The continents on which we live have been moving their locations for millions of years and will continue to move in the future. (True)
9. Human beings, as we know them today, developed from earlier species of animals. (True)
10. Does the Earth go around the Sun, or does the Sun go around the Earth? (Earth around Sun)
11. How long does it take for the Earth to go around the Sun: one day, one month, or one year? (One year)
12. Now, think about this situation. A doctor tells a couple that their genetic makeup means that they've got one in four chances of having a child with an inherited illness. A. Does this mean that if their first child has the illness, the next three will not have the illness? (No)
13. B. Does this mean that each of the couple's children will have the same risk of suffering from the illness? (Yes)
14. Now, please think about this situation. Two scientists want to know if a certain drug is effective against high blood pressure. The first scientist wants to give the drug to one thousand people with high blood pressure and see how many of them experience lower blood pressure levels. The second scientist wants to give the drug to five hundred people with high blood pressure, and not give the drug to another five hundred people with high blood pressure, and see how many in both groups experience lower blood pressure levels. Which is the better way to test this drug? (500 get the drug and 500 don't)

S3. EFA standardized loadings, n = 1,620

Item	Factor 1	Factor2	Communality	Uniqueness
close	0.25	0.17	0.13	0.87
mar	0.73	-0.10	0.48	0.52
live	0.44	-0.02	0.19	0.81
work	-0.05	0.55	0.28	0.72
intl	-0.02	0.48	0.22	0.78

Note: SS loadings: 0.76 (F1) and 0.53 (F2); 26% of the total variance in the items is explained by two factors. The first accounts for 59% of the explained variance while the second accounts for 41%. Correlation of regression scores with factors: 0.76 (F1) and 0.68 (F2). Multiple R2 of scores with factors: 0.57 (F1) and 0.46 (F2). Factor correlation is 0.48. Please see Table 1 in the main body of the paper for item wordings.

S4. Regression models - Without *evolved* and *bigbang* items

	Civic scientific literacy							
	(1)	(2)	(3)	(4)	(5)	(6)	(7)	(8)
Race and Controls								
White	1.658***	1.112***	1.865***	1.871***	1.522***	1.523***	1.223***	1.219***
	(0.104)	(0.120)	(0.204)	(0.208)	(0.214)	(0.215)	(0.223)	(0.223)
Female	-0.538***	-0.724***			-0.517***	-0.535***	-0.655***	-0.669***
	(0.070)	(0.080)			(0.142)	(0.143)	(0.134)	(0.135)
Age	-0.023***	-0.023***			-0.020***	-0.020***	-0.019***	-0.020***
	(0.002)	(0.002)			(0.004)	(0.004)	(0.004)	(0.004)
Independent	-0.089	0.032			0.015	0.016	0.062	0.052
	(0.078)	(0.083)			(0.151)	(0.148)	(0.141)	(0.139)
Republican	0.030	0.176			0.353*	0.320*	0.263*	0.235
	(0.099)	(0.107)			(0.181)	(0.181)	(0.157)	(0.155)
Family income	0.577***	0.134***			0.525***	0.509***	0.131**	0.124*
	(0.039)	(0.045)			(0.066)	(0.066)	(0.063)	(0.064)
Church attendance	-0.016	-0.026*			-0.045	-0.047	-0.036	-0.035
	(0.014)	(0.015)			(0.029)	(0.029)	(0.025)	(0.025)
Education-related								
Education		0.134***					0.165***	0.164***
		(0.020)					(0.032)	(0.032)
College-level science		0.652***					0.516***	0.520***

	Civic scientific literacy							
	(1)	(2)	(3)	(4)	(5)	(6)	(7)	(8)
courses taken								
		(0.114)					(0.174)	(0.175)
Foundational literacy		0.420***					0.407***	0.408***
		(0.022)					(0.044)	(0.044)
Interaction terms								
Racial self-identification			-0.079		0.001		0.003	
			(0.232)		(0.250)		(0.237)	
White x Racial self-identification			-0.328		-0.345		-0.190	
			(0.255)		(0.280)		(0.261)	
Ingroup evaluation				-0.005		0.325		0.345
				(0.247)		(0.235)		(0.229)
White x Ingroup evaluation				-0.549*		-0.704***		-0.563**
				(0.280)		(0.269)		(0.253)
Constant	8.169***	4.146***	6.472***	6.472***	8.011***	8.062***	3.507***	3.561***
	(0.137)	(0.285)	(0.187)	(0.191)	(0.283)	(0.292)	(0.468)	(0.472)
Observations	6,153	3,720	1,620	1,620	1,421	1,421	1,300	1,300
Log Likelihood	-14,339.390	-8,019.526	-3,830.566	-3,826.371	-3,279.158	-3,278.215	-2,773.341	-2,771.273

	Civic scientific literacy							
	(1)	(2)	(3)	(4)	(5)	(6)	(7)	(8)
Akaike Inf. Crit.	28,694.780	16,061.050	7,669.133	7,660.743	6,578.316	6,576.431	5,572.681	5,568.545

Note: *p<0.1; **p<0.05; ***p<0.01. Design-corrected standard errors reported in parentheses. Black is a reference category for race. Male is a reference category for gender. Democrat is a reference category for political preference. Items dropped from the science literacy scale: *evolved:* Human beings, as we know them today, developed from earlier species of animals; *bigbang*: The universe began with a huge explosion.

S5. Regression models – Only among Whites

	Civic scientific literacy							
	(1)	(2)	(3)	(4)	(5)	(6)	(7)	(8)
Controls								
Female	-0.714***	-0.852***			-0.608***	-0.617***	-0.748***	-0.750***
	(0.088)	(0.093)			(0.176)	(0.176)	(0.165)	(0.164)
Age	-0.026***	-0.028***			-0.024***	-0.024***	-0.024***	-0.024***
	(0.003)	(0.003)			(0.005)	(0.005)	(0.005)	(0.005)
Independent	-0.259***	-0.077			-0.238	-0.233	-0.115	-0.114
	(0.100)	(0.107)			(0.193)	(0.191)	(0.175)	(0.174)
Republican	-0.259**	-0.059			0.055	0.023	0.003	-0.019
	(0.119)	(0.137)			(0.226)	(0.226)	(0.201)	(0.197)
Family income	0.677***	0.190***			0.632***	0.614***	0.235***	0.228***
	(0.044)	(0.051)			(0.076)	(0.077)	(0.077)	(0.077)
Church attendance	-0.078***	-0.089***			-0.107***	-0.110***	-0.102***	-0.104***
	(0.017)	(0.018)			(0.034)	(0.034)	(0.028)	(0.028)
Education-related								
Education		0.167***					0.192***	0.191***
		(0.023)					(0.038)	(0.038)
College-level science courses taken		0.721***					0.513***	0.525***
		(0.128)					(0.189)	(0.192)

	Civic scientific literacy							
	(1)	(2)	(3)	(4)	(5)	(6)	(7)	(8)
Foundational literacy		0.469***					0.447***	0.447***
		(0.026)					(0.050)	(0.050)
Racial self-identification			-0.502***		-0.393***		-0.219**	
			(0.113)		(0.122)		(0.107)	
Ingroup evaluation				-0.646***		-0.414***		-0.228**
				(0.131)		(0.129)		(0.112)
Constant	11.138***	5.822***	9.002***	9.010***	10.854***	10.886***	5.419***	5.442***
	(0.168)	(0.347)	(0.086)	(0.088)	(0.316)	(0.319)	(0.556)	(0.549)
Observations	5,147	3,115	1,342	1,342	1,179	1,179	1,075	1,075
Log Likelihood	-12,561.000	-7,012.897	-3,305.804	-3,302.496	-2,828.457	-2,829.501	-2,383.875	-2,384.352
Akaike Inf. Crit.	25,136.000	14,045.790	6,615.607	6,608.992	5,672.913	5,675.002	4,789.750	4,790.705

Note: *p<0.1; **p<0.05; ***p<0.01. Design-corrected standard errors reported in parentheses. Male is a reference category for gender. Democrat is a reference category for political preference.

S6. Regression models – Only among Blacks

	Civic scientific literacy							
	(1)	(2)	(3)	(4)	(5)	(6)	(7)	(8)
Controls								
Female	-0.335	-0.703**			-0.752*	-0.812*	-0.971**	-1.048**
	(0.207)	(0.272)			(0.417)	(0.422)	(0.444)	(0.449)
Age	-0.025***	-0.015**			-0.021	-0.024*	-0.022	-0.025*
	(0.006)	(0.008)			(0.012)	(0.012)	(0.013)	(0.014)
Independent	-0.063	-0.168			0.179	0.181	0.011	-0.023
	(0.201)	(0.245)			(0.463)	(0.444)	(0.446)	(0.429)
Republican	0.299	0.191			0.320	0.182	-0.084	-0.253
	(0.461)	(0.398)			(0.848)	(0.868)	(0.589)	(0.612)
Family income	0.746***	0.109			0.568*	0.561*	-0.101	-0.103
	(0.122)	(0.145)			(0.283)	(0.280)	(0.306)	(0.302)
Church attendance	-0.120***	-0.154***			-0.106	-0.101	-0.090	-0.081
	(0.037)	(0.047)			(0.081)	(0.080)	(0.081)	(0.080)
Education-related								
Education		0.094*					0.123	0.121
		(0.055)					(0.109)	(0.107)
College-level science courses taken		0.594*					0.584	0.583
		(0.328)					(0.571)	(0.559)

	Civic scientific literacy							
	(1)	(2)	(3)	(4)	(5)	(6)	(7)	(8)
Foundational literacy		0.311***					0.316***	0.319***
		(0.071)					(0.104)	(0.104)
Racial self-identification			-0.114		-0.005		0.032	
			(0.260)		(0.270)		(0.262)	
Ingroup evaluation				-0.123		0.273		0.330
				(0.261)		(0.243)		(0.254)
Constant	9.083***	5.982***	6.953***	6.945***	8.900***	9.059***	5.467***	5.676***
	(0.367)	(0.739)	(0.220)	(0.221)	(0.762)	(0.766)	(1.360)	(1.355)
Observations	1,006	605	278	278	242	242	225	225
Log Likelihood	-2,430.766	-1,419.017	-702.928	-702.908	-581.056	-580.228	-529.478	-528.321
Akaike Inf. Crit.	4,875.532	2,858.033	1,409.855	1,409.817	1,178.112	1,176.455	1,080.956	1,078.641

Note: *p<0.1; **p<0.05; ***p<0.01. Design-corrected standard errors reported in parentheses. Male is a reference category for gender. Democrat is a reference category for political preference.

S7. Regression models - Including *science attitudes* as a predictor

	Civic scientific literacy							
	(1)	(2)	(3)	(4)	(5)	(6)	(7)	(8)
Race and Controls								
White	1.905***	1.289***	2.048***	2.065***	1.687***	1.694***	1.420***	1.400***
	(0.116)	(0.169)	(0.237)	(0.238)	(0.228)	(0.228)	(0.264)	(0.261)
Female	-0.660***	-0.763***			-0.626***	-0.642***	-0.562***	-0.567***
	(0.079)	(0.095)			(0.156)	(0.157)	(0.161)	(0.163)
Age	-0.026***	-0.025***			-0.024***	-0.024***	-0.022***	-0.022***
	(0.002)	(0.003)			(0.004)	(0.004)	(0.004)	(0.005)
Independent	-0.212**	0.009			-0.156	-0.152	-0.004	-0.001
	(0.088)	(0.106)			(0.172)	(0.169)	(0.181)	(0.178)
Republican	-0.207*	0.023			0.107	0.070	0.090	0.053
	(0.110)	(0.131)			(0.211)	(0.212)	(0.198)	(0.195)
Family income	0.682***	0.155***			0.625***	0.608***	0.236***	0.225***
	(0.042)	(0.048)			(0.072)	(0.072)	(0.079)	(0.079)
Church attendance	-0.084***	-0.099***			-0.107***	-0.109***	-0.087***	-0.089***
	(0.015)	(0.018)			(0.032)	(0.031)	(0.029)	(0.029)
Education-related								
Education		0.143***					0.188***	0.187***
		(0.022)					(0.040)	(0.040)
College-level science courses taken		0.651***					0.381*	0.385*
		(0.133)					(0.218)	(0.220)
Foundational literacy		0.418***					0.393***	0.395***
		(0.028)					(0.049)	(0.049)

	Civic scientific literacy							
	(1)	(2)	(3)	(4)	(5)	(6)	(7)	(8)
Positive science attitude		0.877***					0.777***	0.800***
		(0.184)					(0.257)	(0.252)
Interaction terms								
Racial self-identification			-0.114		-0.022		0.011	
			(0.260)		(0.264)		(0.292)	
White x Racial self-identification			-0.388		-0.369		-0.328	
			(0.280)		(0.292)		(0.313)	
Ingroup evaluation				-0.123		0.256		0.381
				(0.261)		(0.242)		(0.325)
White x Ingroup evaluation				-0.523*		-0.668**		-0.726**
				(0.294)		(0.272)		(0.338)
Constant	9.186***	4.326***	6.953***	6.945***	9.108***	9.153***	3.484***	3.542***
	(0.155)	(0.368)	(0.220)	(0.221)	(0.321)	(0.324)	(0.572)	(0.577)
Observations	6,153	2,921	1,620	1,620	1,421	1,421	1,011	1,011
Log Likelihood	-14,999.790	-6,556.369	-4,007.740	-4,004.421	-3,410.107	-3,410.441	-2,239.715	-2,238.813
Akaike Inf. Crit.	30,015.580	13,136.740	8,023.479	8,016.842	6,840.215	6,840.883	4,507.429	4,505.625

Note: *p<0.1; **p<0.05; ***p<0.01. Design-corrected standard errors reported in parentheses. Black is a reference category for race. Male is a reference category for gender. Democrat is a reference category for political preference. Negative science attitude is a reference category for positive science attitude. Science attitude is measured with *balpos* and *balneg* items of the GSS. Those considered as having a positive attitude answered ‘Strongly in favor’ or ‘Slightly in favor’ on the question about the benefits of scientific research outweighing their harmful results (*balpos*). Those considered as having a negative attitude answered ‘Strongly in favor’ or ‘Slightly in favor’ on the question about the harmful results of scientific research outweighing their benefits (*balneg*). 91,5% of whites report having positive attitude to science, whereas this number is 77.8% for Blacks.

S8. Regression models - Including squared terms

	Civic scientific literacy							
	(1)	(2)	(3)	(4)	(5)	(6)	(7)	(8)
Race and Controls								
White	1.845***	1.295***	2.021***	1.994***	1.611***	1.607***	1.375***	1.383***
	(0.117)	(0.137)	(0.237)	(0.240)	(0.240)	(0.241)	(0.245)	(0.243)
Female	-0.651***	-0.829***			-0.610***	-0.641***	-0.748***	-0.763***
	(0.079)	(0.090)			(0.155)	(0.155)	(0.152)	(0.151)
Age	0.008	0.008			-0.019	-0.017	-0.004	-0.002
	(0.012)	(0.014)			(0.024)	(0.024)	(0.022)	(0.022)
Age^2	-0.0003***	-0.0004***			-0.00005	-0.0001	-0.0002	-0.0002
	(0.0001)	(0.0001)			(0.0002)	(0.0002)	(0.0002)	(0.0002)
Independent	-0.213**	-0.111			-0.189	-0.180	-0.113	-0.124
	(0.087)	(0.093)			(0.168)	(0.165)	(0.158)	(0.156)
Republican	-0.233**	-0.046			0.082	0.034	0.011	-0.024
	(0.109)	(0.126)			(0.208)	(0.209)	(0.189)	(0.189)
Family income	1.013***	0.248***			0.926***	0.899***	0.314***	0.306***
	(0.065)	(0.068)			(0.125)	(0.125)	(0.110)	(0.112)
Family income^2	-0.212***	-0.047			-0.207***	-0.202***	-0.072	-0.072
	(0.031)	(0.031)			(0.060)	(0.060)	(0.053)	(0.053)
Church attendance	-0.078	-0.112*			-0.194**	-0.215**	-0.223**	-0.232***
	(0.051)	(0.059)			(0.098)	(0.099)	(0.086)	(0.087)
Church attendance^2	-0.001	0.002			0.011	0.014	0.017	0.018
	(0.006)	(0.007)			(0.012)	(0.012)	(0.011)	(0.011)
Education-related								
Education		0.212**					0.387**	0.386**
		(0.103)					(0.188)	(0.184)

	Civic scientific literacy							
	(1)	(2)	(3)	(4)	(5)	(6)	(7)	(8)
Education^2		-0.002					-0.008	-0.008
		(0.004)					(0.007)	(0.007)
College-level science courses taken		0.735***					0.551***	0.559***
		(0.127)					(0.194)	(0.196)
Foundational literacy		0.208**					-0.018	-0.014
		(0.093)					(0.167)	(0.166)
Foundational literacy^2		0.020***					0.037***	0.037***
		(0.008)					(0.012)	(0.012)
Interaction terms								
Racial self-identification			-0.144		-0.040		-0.001	
			(0.253)		(0.262)		(0.264)	
Racial self-identification^2			-0.315**		-0.165		-0.037	
			(0.139)		(0.148)		(0.135)	
White x Racial self-identification			-0.368		-0.348		-0.223	
			(0.272)		(0.287)		(0.280)	
Ingroup evaluation				-0.034		0.334		0.312
				(0.251)		(0.244)		(0.254)
Ingroup evaluation^2				-0.333***		-0.163		0.037
				(0.111)		(0.121)		(0.103)
White x Ingroup evaluation				-0.492*		-0.671**		-0.566**
				(0.286)		(0.266)		(0.266)
Constant	8.683***	4.413***	7.155***	7.159***	9.435***	9.445***	3.986***	3.932***
	(0.330)	(0.798)	(0.241)	(0.237)	(0.659)	(0.643)	(1.441)	(1.401)
Observations	6,153	3,720	1,620	1,620	1,421	1,421	1,300	1,300
Log Likelihood	-14,955.230	-8,435.819	-4,003.922	-3,998.636	-3,400.910	-3,400.982	-2,910.447	-2,909.290

	Civic scientific literacy							
	(1)	(2)	(3)	(4)	(5)	(6)	(7)	(8)
Akaike Inf. Crit.	29,932.450	16,903.640	8,017.843	8,007.273	6,829.821	6,829.964	5,858.894	5,856.579

Note: *p<0.1; **p<0.05; ***p<0.01. Design-corrected standard errors reported in parentheses. Black is a reference category for race. Male is a reference category for gender. Democrat is a reference category for political preference.

S9. Regression models – Using the Difference measure of racial self-identification and ingroup-evaluation*

	Civic scientific literacy							
	(1)	(2)	(3)	(4)	(5)	(6)	(7)	(8)
Race and Controls								
White	1.905***	1.283***	2.122***	1.999***	1.593***	1.529***	1.250***	1.189***
	(0.116)	(0.135)	(0.285)	(0.317)	(0.274)	(0.288)	(0.292)	(0.302)
Female	-0.660***	-0.837***			-0.703***	-0.711***	-0.812***	-0.803***
	(0.079)	(0.089)			(0.153)	(0.153)	(0.150)	(0.152)
Age	-0.026***	-0.026***			-0.022***	-0.020***	-0.023***	-0.022***
	(0.002)	(0.002)			(0.004)	(0.004)	(0.004)	(0.004)
Independent	-0.212**	-0.099			-0.119	-0.117	-0.065	-0.061
	(0.088)	(0.093)			(0.169)	(0.170)	(0.158)	(0.160)
Republican	-0.207*	-0.034			0.134	0.187	0.037	0.055
	(0.110)	(0.125)			(0.210)	(0.213)	(0.185)	(0.186)
Family income	0.682***	0.184***			0.577***	0.581***	0.196***	0.196***
	(0.042)	(0.047)			(0.071)	(0.072)	(0.073)	(0.073)
Church attendance	-0.084***	-0.099***			-0.107***	-0.111***	-0.098***	-0.099***
	(0.015)	(0.017)			(0.031)	(0.031)	(0.028)	(0.029)
Education-related								
Education		0.157***					0.173***	0.177***
		(0.022)					(0.035)	(0.035)
College-level science courses taken		0.714***					0.524**	0.511**

	Civic scientific literacy							
	(1)	(2)	(3)	(4)	(5)	(6)	(7)	(8)
		(0.125)					(0.202)	(0.200)
Foundational literacy		0.444***					0.421***	0.424***
		(0.025)					(0.047)	(0.047)
Interaction terms								
DIFF. Racial self-identification			-0.086		0.174		0.170	
			(0.181)		(0.163)		(0.184)	
White x DIFF. Racial self-identification			-0.519***		-0.719***		-0.436**	
			(0.197)		(0.174)		(0.194)	
DIFF. Ingroup evaluation				0.155		0.329		0.356
				(0.309)		(0.292)		(0.307)
White x DIFF. Ingroup evaluation				-0.837***		-0.920***		-0.600*
				(0.318)		(0.302)		(0.307)
Constant	9.186***	4.760***	6.953***	7.072***	9.179***	9.159***	4.547***	4.510***
	(0.155)	(0.316)	(0.266)	(0.298)	(0.366)	(0.362)	(0.534)	(0.546)
Observations	6,153	3,720	1,603	1,603	1,409	1,409	1,288	1,288
Log Likelihood	-14,999.790	-8,445.311	-3,942.619	-3,943.390	-3,364.856	-3,367.448	-2,884.863	-2,886.592
Akaike Inf. Crit.	30,015.580	16,912.620	7,893.238	7,894.779	6,749.711	6,754.897	5,795.725	5,799.185

Note: $^{*}p<0.1$; $^{**}p<0.05$; $^{***}p<0.01$. Design-corrected standard errors reported in parentheses. Black is a reference category for race. Male is a reference category for gender. Democrat is a reference category for political preference. *The Difference measures of racial self-identification and ingroup evaluation are computed by subtracting the values obtained for the outgroup race from the values obtained for the ingroup race e.g. for 1) Whites: Whites – Blacks and for 2) Blacks: Blacks – Whites.
